# Circular RNAs in hepatocellular carcinoma: Functions and implications

**DOI:** 10.1002/cam4.1574

**Published:** 2018-06-01

**Authors:** Liyun Fu, Zhenluo Jiang, Tianwen Li, Yaoren Hu, Junming Guo

**Affiliations:** ^1^ Department of Hepatology Ningbo No. 2 Hospital, and the Affiliated Hospital Medical School of Ningbo University Ningbo China; ^2^ Department of Biochemistry and Molecular Biology Zhejiang Key Laboratory of Pathophysiology Medical School of Ningbo University Ningbo China

**Keywords:** circular RNA, function, hepatocellular carcinoma, microRNA, sponge

## Abstract

At present, as hotspot members of the noncoding RNA network, circular RNAs (circRNAs) with distinct properties and diverse pathophysiological functions are being increasingly delineated. CircRNAs play roles at the epigenetic, transcriptional and posttranscriptional regulatory levels. Major studies have focused on their functions as efficient microRNA sponges. The validated number of endogenous circRNAs involved in hepatocellular carcinoma (HCC) continues to increase. Altered circRNA expression is associated with HCC occurrence, invasion, and metastasis. Moreover, the aberrant expression of circRNAs is also significantly related to HCC tumor stage, size, differentiation and metastasis. Because they are exceptionally stable, highly conserved and have tissue‐specific expression patterns, some circRNAs, including hsa_circ_0004018, hsa_circ_0003570, and hsa_circ_0005075, may be potential markers for the diagnosis of HCC. We herein summarize the current knowledge of HCC‐associated circRNAs and present their implications for carcinogenesis and their potential value as diagnostic and prognostic biomarkers. Finally, we discuss the future directions of studies on HCC‐associated circRNAs.

## 1 INTRODUCTION

Circular RNAs (circRNAs) refer to a large class of RNA species without 3′ or 5′ ends and that are widely expressed in eukaryotic cells.^1^, ^2^, ^3^ For several decades, most circRNAs were considered to be inconspicuous alternative splicing transcripts. However, recent advances in large‐scale deep sequencing have revealed the widespread prevalence of circRNAs. In contrast to conventional linear splicing RNAs, circRNAs are featured by ‘back‐splicing’ events, which endow them the properties of exonuclease resistance, long half‐life and inherent stability. Now, an increasing number of studies has demonstrated that circRNAs play important biological roles in the normal development of tissues or organs and in the occurrence and pathogenesis of diseases.^1^, ^2^, ^3^

Hepatocellular carcinoma (HCC) is a highly heterogeneous malignancy derived from intricate genetic and epigenetic alterations and is the second leading cause of cancer‐related death worldwide. When diagnosed at the early stage, HCC patients can achieve marked advances in life expectancy; however, when diagnosed at the advanced stage with metastasis, patients have dismal prognoses, even if they are treated with multikinase inhibitor sorafenib or regorafenib.^4^ The challenges confronting hepatologists worldwide are mainly focused on how to screen HCC patients at the earlier stage and thereby perform timely curative procedures, such as radical primary resection, ablation or liver transplantation. Therefore, exploring the molecular mechanism and identifying valuable markers of HCC are extremely important.

## 2 BIOGENESIS OF CIRCRNAS

**Figure 1:**
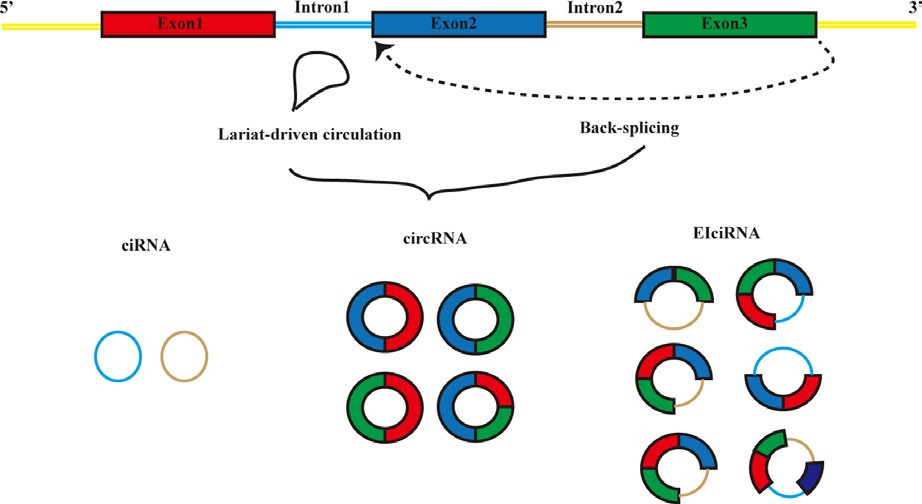
The biogenesis of circular RNA (circRNA). Circular RNAs are generated from lariat‐driven circulation or back‐splicing events between the splice donor site of a downstream exon and the splice acceptor site of an upstream exon

CircRNAs are formed through lariat‐circulating or back‐splicing events from all regions of the genome and are derived mostly from exons; however, in rare cases, circRNAs are derived from intergenic, intronic regions, antisense or untranslated region (UTR) regions. The terms circRNA, EIciRNA and ciRNA are used to describe circRNAs originating from the exon, exon and intron, and intron, respectively (Figure 1). CircRNAs can be found in most subcellular compartments, but the majority are localized predominantly in the cytoplasm.^1^, ^2^, ^3^, ^5^ CircRNA formation is influenced by several sequence features, such as intron length, exon length, repetitive sequences, and RNA‐binding proteins (RBPs), including quaking (QKI), adenosine deaminases that act on RNA (ADAR1), NF90/NF110, heterogeneous nuclear ribonucleoprotein L (HNRNPL) and muscleblind (MBL/MBNL1).^6^, ^7^, ^8^, ^9^, ^10^, ^11^ Recently, based on RNA interference screening, Liang et al^12^ revealed that the expression ratio of linear to circular RNA is modulated by many core spliceosomal and transcription termination factors.

**Figure 2:**
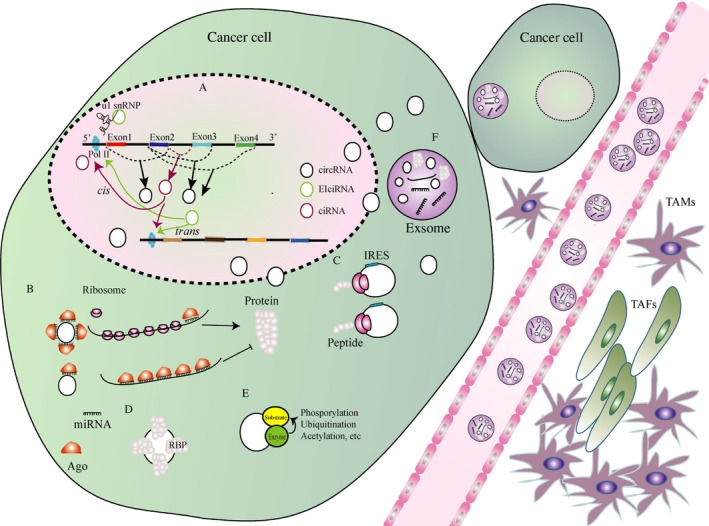
Function of circRNAs. A, ciRNAs interact with RNA Pol II, and EIciRNAs associate with RNA Pol II and U1 snRNP and thereby enhance the transcription of their parental genes. B, Most circRNAs can act as miRNA sponges or sequesterers. C, CircRNAs with IRES elements might be used as translational templates. D, CircRNAs with RBP motifs may function as sponges or decoys for proteins and thereby regulate their activity. E, CircRNAs with binding motifs for an enzyme and its substrate may function as scaffolds facilitating co‐localization and reaction kinetics. F, Exosomes containing circRNAs are secreted from cells into blood vessels or mediate cell‐cell communication. Abbreviations: IRES: Internal ribosome entry site, TAMs: tumor associated macrophages, TAFs: tumor associated fibroblasts

How are circRNAs degraded or cleared? Scientists have recently focused on exosomes. Exosomes, which are double‐membrane vehicles containing different functional molecules, such as proteins, microRNAs (miRNAs), long noncoding RNAs (lncRNAs), and circRNAs, may mediate cell‐cell communication in a paracrine or endocrine manner^13^ (Figure 2). The expulsion of circRNAs through exosomal release might be an effective way to clear or degrade circRNAs.^14^

## 3 FUNCTIONS OF CIRCRNAS

### 3.1 Regulation of transcription

Intron‐containing circRNAs, such as ciRNAs and EIciRNAs, are abundant in the nucleus. How ciRNAs and EIciRNAs are restricted to the nucleus remains vague. These circRNAs may regulate the expression of their host genes in either a *cis*‐acting manner or a *trans*‐acting manner (Figure 2A). Some ciRNAs, such as ci‐ankrd52 and ci‐sirt7, can gather at the transcriptional sites of host genes, interact with the RNA polymerase II (Pol II) complex, and act as positive regulators of parental gene transcription.^15^ Li et al^16^ found that EIciEIF3J and EIciPAIP2 can interact with the Pol II complex and U1 small nuclear ribonucleoproteins (snRNP) and thereby increase Eukaryotic translation initiation factor 3 subunit J (*EIF3J)* and Polyadenylate‐binding protein‐interacting protein 2 (*PAIP2)* transcription in a *cis*‐acting manner.

### 3.2 MiRNA sequestration

CircRNAs harbor well‐conserved canonical miRNA response elements (MREs), suggesting that some circRNAs act as miRNA sequesters or sponges.^17^, ^18^, ^19^, ^20^ A portion of circRNAs are thought to negatively regulate the levels of miRNAs and increase or decrease the expression levels of their corresponding targeted genes (Figure 2B). As a representative circRNA, cerebellar degeneration‐related protein 1 antisense (*CDR1as*) is the sponge of miR‐7 in the embryonic zebrafish and has been reported to be related to brain dysfunction and many cancers, such as HCC and gastric cancer.^21^, ^22^, ^23^, ^24^ Accordingly, an increasing number of studies has revealed that the circRNA‐miRNA‐target gene regulatory network might have profound implications for understanding diseases, especially cancers.^25^, ^26^ For example, a recent study shows that circ‐ZNF609 acts as an endogenous miR‐615‐5p sponge to inhibit miR‐615‐5p activity, increasing myocyte‐specific enhancer factor 2A (MEF2A) expression and ameliorating vascular endothelial dysfunction.^27^ Zhong et al^28^ found that circ‐MYLK is not only related to the stage and grade of the cancer but can also work with miR‐29a and relieve the suppression of target vascular endothelial growth factor A (*VEGFA*), a key member of the VEGFA/VEGFR2/Ras/ERK signaling pathway. Increased circ‐UBAP2 promotes cancer cell growth and inhibits apoptosis both in vitro and in vivo by sequestering miR‐143, thus enhancing its target antiapoptotic *Bcl‐2* expression.^29^ Liang et al^30^ disclosed that circ‐ABCB10 can sponge miR‐1271, increasing proliferation and decreasing apoptosis. Hsa_circ_000984 derived from a cell division protein kinase (*CDK6*) may act as a competing endogenous RNA (ceRNA) by sequestering miR‐106b.^31^ Circ‐HIPK2 derived from *HIPK2* gene exon 2 can affect the distinct roles of its target in cell autophagy by sponging miR‐124 (miR124‐2HG), thus regulating sigma nonopioid intracellular receptor 1 (*SIGMAR1/OPRS1*) expression in astrocyte cells.^32^ However, circ‐HIPK3 derived from *HIPK3* gene exon 2 can abundantly sponge miR‐558 to suppress the expression of heparanase (*HPSE*) in bladder cancer and can sponge endogenous miR‐30a‐3p in diabetes mellitus‐associated retinal vascular dysfunction.^33^, ^34^

### 3.3 Templates for translation

As most circRNAs originate from exons, are located in the cytoplasm, might contain internal ribosome entry site (IRES), and include an open reading frame (ORF), they may be efficiently translated^35^, ^36^ (Figure 2C). Yang et al validated that some circRNAs are rich in N6‐methyladenosine (m6A) motifs that enable translation initiation. This study revealed that thousands of endogenous circRNAs might have translation ability.^37^ Yang et al^38^ validated that circ‐FBXW7 contains an ORF driven by IRES and translates into a novel 21‐kDa protein (termed FBXW7‐185aa). Legnini et al^39^ identified an ORF‐containing circ‐ZNF609 translated into a protein in a splicing‐dependent/cap‐independent manner in human and murine myoblasts.

### 3.4 CircRNA‐protein interaction

CircRNAs may be used to bind, store, or sequester protein molecules. Most circRNAs may interact with RBPs (Figure 2D). With a high density of binding sites for a single or multiple RBP, some circRNAs may function as protein sponges or decoys. EIciRNAs and ciRNAs promote the transcription of their parental genes by interacting with host U1 snRNP and/or RNA Polymerase II. The best experimentally supported example of a circRNA interacting with a protein is *CDR1as*, which is associated with Argonaute (AGO) proteins in a miR‐7‐dependent manner.^21^, ^40^ Coon et al^6^ validated that the circRNA abundance decreases upon *QKI* knockdown and fluctuates during the human epithelial‐mesenchymal transition (EMT) process. The circRNA derived from the *MBL* locus (circ‐MBL) harbors binding sites for the MBL protein itself and prevents MBL protein from binding to other targets when tethered to the circ‐MBL.^8^ In additional, the circRNA derived from the *PABPN1* locus (circ‐PABPN1) harbors binding sites for ELAV‐like protein 1 (HuR) and hence prevents HuR from binding to *PABPN1* mRNA and lowers its translation.^41^ In contrast to the vast majority of exon‐derived circRNAs that are located in the cytoplasm, circ‐Amotl1 (hsa_circ_0004214) tends to interact with and stabilize oncogene *C‐MYC* in the nucleus. Hsa_circ_0004214 enhances *C‐MYC* binding to the promoters of hypoxia‐inducing factor (HIF)‐1α, cell division cycle 25 homolog A (Cdc25a), ETS domain‐containing protein (ELK1), and JUN, thus promoting cell proliferation, invasion and colony formation.^42^

### 3.5 Scaffold for enzymes

Those circRNAs that harbor binding domains for enzymes and their substrates are likely to function as scaffolds to connect two or more proteins (Figure 2E). To date, circ‐Foxo3 is the best example of displaying a variety of tertiary structures in various cell/tissue environments.^43^ In the mouse fibroblast NIH3T3 cell line, circ‐Foxo3 can bind to cyclin‐dependent kinase inhibitor 1 (p21) and cyclin‐dependent kinase 2 (CDK2), and this binding represses cell cycle entry.^44^ Mouse double minute 2 homolog (*MDM2*) is an important negative regulator of the *p53* tumor suppressor, and MDM2 protein functions as both an E3 ubiquitin ligase that recognizes the N‐terminal transactivation domain (TAD) of the *p53* tumor suppressor and as an inhibitor of *p53* transcriptional activation. Circ‐Foxo3 can bind to MDM2 and p53, promote MDM2‐induced p53 ubiquitination and subsequent degradation, and thus induce tumor cell apoptosis.^45^ In addition, circ‐Foxo3 can bind to stress‐related proteins and transcription factors, such as DNA‐binding protein inhibitor (ID‐1), E2F1, focal adhesion kinase (FAK), and HIF1α, thus promoting cardiac senescence.^46^

## 4 CIRCRNAS AND HCC

In accordance with the pivotal role in epigenetic and genetic regulation mentioned above, the deregulation of circRNA expression has been reported in several diseases, such as cancers, neurological disorders, heart disease, diabetes, and atherosclerosis.^40^, ^47^, ^48^, ^49^, ^50^, ^51^, ^52^ In additional, the hypothesis of the intertwining of HCC with circRNAs is being validated.

### 4.1 CircRNAs as reliable biomarkers for HCC

The early diagnosis of HCC in patients particularly is important. Due to their complex tissue‐ or cell‐type and developmental stage‐specific patterns, resistance to ribonucleases, such as exonucleases and RNase R, and longer half‐lives, circRNAs are presumed to be ideal biomarkers.^15^, ^16^, ^18^, ^19^, ^53^, ^54^, ^55^ Based on microarray screening, our recent study identified 527 differentially expressed circRNAs; hsa_circ_0005986, hsa_circ_0003570, and hsa_circ_0004018 were significantly downregulated in HCC samples compared to adjacent noncancerous tissues and had the ability to differentiate HCC from liver cirrhosis and chronic hepatitis.^56^, ^57^, ^58^ Other groups have found that circ‐ZKSCAN1 is significantly downregulated in HCC samples compared with adjacent nontumorous tissues and is related to tumor number, cirrhosis, vascular invasion, and microscopic vascular invasion as well as tumor grade.^59^ Shang et al^60^ reported that hsa_circ_0005075 is upregulated in HCC samples compared to adjacent noncancerous tissues and is involved in HCC development. Han et al^61^ revealed that hsa_circ_0007874 is downregulated in HCC and associated with poor patient survival. Hsa_circ_0001649 derived from the *SHPRH* gene is significantly downregulated in HCC samples and correlated with tumor size and the occurrence of tumor embolus.^62^ Yu et al^63^ revealed that the downregulation of hsa_circ_0001445 in HCC tissues is significantly correlated with aggressive characteristics and may serve as an independent risk factor for overall survival (OS) and recurrence‐free survival (RFS) in HCC patients after hepatectomy.

Single‐nucleotide polymorphisms (SNPs) may affect the susceptibility and clinical outcome of HCC. Guo et al^64^ have found that rs10485505 and rs4911154 in circ‐ITCH are significantly associated with increased HCC risk and can serve as susceptibility and prognostic biomarkers for HCC patients.

Exosomes may serve as good candidates for liquid biopsy, particularly for monitoring and predicting tumor occurrence and metastasis.^13^, ^14^ Li et al^50^ found that circRNAs are abundant and stable in exosomes and that exosomal circRNAs might be a novel kind of promising biomarker for cancer diagnosis. The exosomal circRNAs associated with HCC need to be further deciphered.

### 4.2 CircRNAs in HCC developmental mechanisms

**Figure 3:**
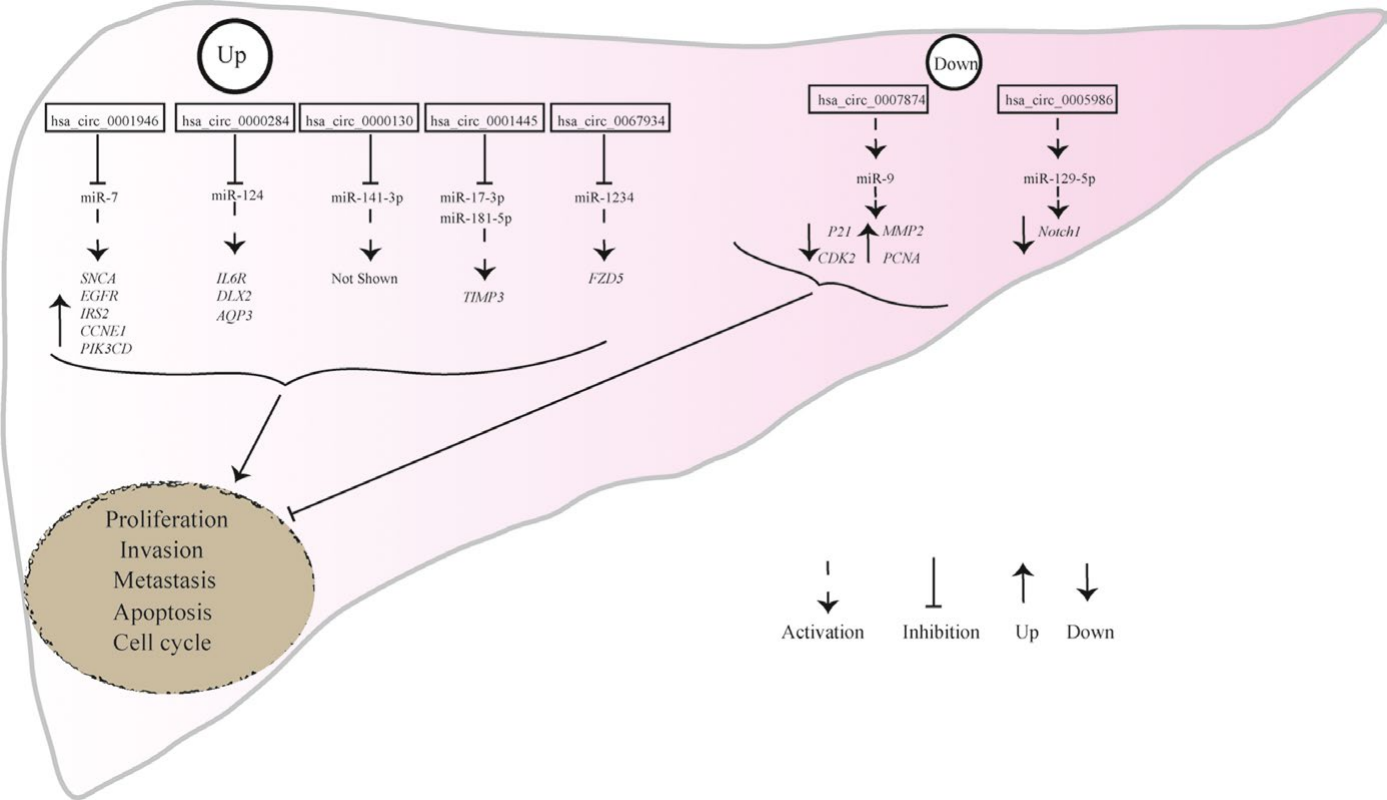
The validated circRNA‐miRNA‐target gene axis. Upregulated circRNAs can promote tumor progression by sequestering tumor‐suppressor miRNAs because sequestration of these miRNAs alleviates the expression of oncogenic targets. Downregulated circRNAs may exert an anti‐tumoral effect when they sponge miRNAs that suppress tumor‐suppressor genes. SNCA, alpha‐synuclein; EGFR, Epidermal growth factor receptor; IRS2, Insulin receptor substrate 2; CCNE1, G1/S‐specific cyclin‐E1; PIK3CD, phosphoinositide 3‐kinase delta isoform; IL6R, Interleukin 6 receptor; DLX2, Distalless 2; AQP3, Aquaporin 3; TIMP3, Metalloproteinase inhibitor 3; FZD5, Frizzled‐5; P21, Cyclin‐dependent kinase inhibitor 1; CDK2, Cyclin‐dependent kinase 2; MMP2, Matrix metalloproteinase‐2; PCNA, Proliferating cell nuclear antigen

As with other tumors, in HCC, an increasing number of studies has shown that circRNAs are good candidates for miRNA sequestration and the subsequent fine‐tuning of target gene expression.^65^, ^66^ To date, most mechanistic studies of HCC have focused on miRNA sponges. Huang et al^65^ revealed that hsa_circ_0000130 (hsa_circ_100338) functions as an endogenous sponge for miR‐141‐3p in the regulation of HCC invasion. Through a quantitative proteomics strategy, Yang et al^66^ showed that epidermal growth factor receptor (EGFR), a validated miR‐7 target, is present in *CDR1as*‐overexpressing HepG2 cells. Our team has reported that hsa_circ_0005986 functions as an effective sponge for miR‐129‐5p, thereby regulating *Notch1* expression.^58^ Acting as a sponge for miR‐9, hsa_circ_0007874 promotes p21 expression and then suppresses HCC progression, suggesting that hsa_circ_0007874 is a potential target for HCC treatment.^61^ Based on RNA‐sequence technology, Zheng et al^48^ discovered that circ*HIPK3* (hsa_circ_0000284) is significantly upregulated in liver cancer compared with matched normal tissues and directly binds to miR‐124, thereby upregulating Interleukin 6 receptor (IL6R) and Homeobox protein DLX2 expression. Chen et al^67^ discovered that hsa_circ_0000284 promotes HCC proliferation and migration by directly binding to miR‐124, thereby upregulating aquaporin 3 (AQP3) expression. By sponging miR‐17‐3p and miR‐181b‐5p, hsa_circ_0001445 promotes the expression of metalloproteinase inhibitor 3 (TIMP3) and then inhibits the proliferation and migration of HCC cells.^63^ Zhu et al^68^ demonstrated that hsa_circ_0067934 enhances the proliferation, migration and invasion of HCC cells via the sponging of miR‐1324 and concomitant activation of the FZD5/Wnt/β‐catenin signaling pathway. The validated circRNA‐miRNA‐target gene axis is shown in Figure 3.

Characterized by mitochondrial dysfunction, growth arrest, and apoptosis, fatty liver disease is a high‐risk factor for HCC. Gou et al^69^ revealed that the circ_0046367‐miR‐34a‐peroxisome proliferator‐activated receptor α axis underlies hepatic steatosis (Table 1).

**Table 1:** Overview of the identified hepatocellular carcinoma‐associated circRNAs

Circbase ID (*Alias*)	Chromosome	Strand	Gene symbol	Function	Expression change	Possible mechanism	References
hsa_circ_0001946 (ciRS‐7)	chrX	+	*CDR1AS*	Promotes cell proliferation and invasion	Up	miRNA sponge	22, 23, 66
has_circ_0001649	chr6	−	*SHPRH*	Inhibits cell proliferation	Down	miRNA sponge^a^	62
hsa_circ_0001727	chr7	+	*ZKSCAN1*	Inhibits cell proliferation, migration, and invasion	Down	miRNA sponge^a^	59
hsa_circ_0005075	chr1	−	*EIF4 gG3*	Promotes cell adhesion	Up	miRNA sponge^a^	60
hsa_circ_0000284	chr11	+	*HIPK3*	Promotes cell proliferation	Up	miRNA sponge	48, 67
hsa_circ_0007874	chr6	_+_	*MTO1*	Inhibits cell proliferation and invasion; promotes apoptosis	Down	miRNA sponge	61
hsa_circ_0004018	chr17	−	*SMYD4*	Not investigated	Down	miRNA sponge^a^	56
hsa_circ_0003570	chr10	−	*FAM53B*	Not investigated	Down	Not investigated	57
hsa_circ_0005986	chr1	+	*PRDM2*	Inhibits cell proliferation and cell cycle progression	Down	miRNA sponge	58
hsa_circ_0000130	chr1	+	*SNX27*	Promotes cell invasion	Up	miRNA sponge	65
hsa_circ_0085154	chr8	−	*ARSP91*	Inhibits colony formation and tumor growth	Down	Regulated by *ADAR1*	7
hsa_circ_0001445	chr4	+	*SMARCA5*	Inhibits proliferation and migration	Down	miRNA sponge	63
hsa_circ_0067934	chr3	+	*PRKCI*	Promotes tumor growth and metastasis	Up	miRNA sponge	68
hsa_circ_0001141	Chr20	+	*ITCH*	Not investigated	Down	Single‐nucleotide polymorphism	64

a Prediction based on bioinformatics, not validated.

### 4.3 CircRNAs in HCC epithelial‐mesenchymal transition

The epithelial‐mesenchymal transition (EMT) is defined as the process although which epithelial cells lose their differentiated properties and obtain mesenchymal characteristics. EMT plays important roles in HCC occurrence and progression.^70^ EMT is regulated by complex transcriptional and posttranscriptional mechanisms.^71^ *CDR1as*, a risk factor of hepatic microvascular invasion, is inversely correlated with the activities of miR‐7.^22^, ^23^, ^66^ MiR‐7 downregulates the expression of EMT‐associated signaling molecules, including EGF receptor (EGFR), insulin‐like growth factor 1 (IGF1) and FAK, thereby indirectly upregulating E‐cadherin and downregulating N‐cadherin expression.^21^ In view of the abovementioned miRNA sponge theory, we can deduce that *CDR1as* might be involved in the EMT process of HCC.

### 4.4 CircRNAs as potential therapeutic targets in HCC

As mentioned above, circRNAs are intimately correlated with HCC carcinogenesis. What is the goal of circRNA application in HCC treatment? Hepatitis C virus (HCV) is an important latent carcinogenic factor for HCC. MiR‐122 is a validated dispensable molecule for the HCV cell‐life cycle. In recent times, Jost et al^72^ successfully constructed artificial circRNAs containing an array of miRNA‐122‐binding sites, and these engineered circRNAs could not only sponge miRNA‐122 and inhibit HCV replication but also affect HCV translation. Taken together, the involvement of circRNAs and miRNAs in HCC provide experimental evidence that circRNAs may serve as novel therapeutic targets for HCC in the future.

### 4.5 Prospect

An increasing number of circRNAs is involved in the deregulation of cancer pathophysiological processes, and these circRNAs have shown great potential in cancer diagnosis, prognosis, and therapy.^73^ We can utilize plasmids to increase the expression of eukaryotic circRNAs and CRISPR/Cas9 or siRNA techniques to decrease circRNA expression.^74^ The RT‐droplet digital PCR (RT‐ddPCR) method, a potent noninvasive and absolute quantification method, has been utilized to detect circRNA expression.^75^ Moreover, to avoid tumor heterogeneous interference, we can use single‐cell sequencing analysis to reveal the circRNA profile at the single‐cell level.^76^ The cancer‐specific circRNA database (CSCD, http://gb.whu.edu.cn/CSCD) is a comprehensive database for exploring cancer‐specific circRNAs.^77^ CSCD will enable studies of the functions of cancer‐associated circRNAs by predicting MRE sites, RBP sites and potential ORFs. At last, deregulated circRNAs not only facilitate the understanding of the complex etiology of HCC but also promise to be a kind of ideal biomarker.

## CONFLICT OF INTEREST

None declared.
